# Survey and characterization of nonfunctional alleles of *FUT2* in a database

**DOI:** 10.1038/s41598-021-82895-w

**Published:** 2021-02-04

**Authors:** Mikiko Soejima, Yoshiro Koda

**Affiliations:** Department of Forensic Medicine, Kurume University School of Medicine, Kurume, 830-0011 Japan

**Keywords:** Biochemistry, Genetics, Molecular medicine

## Abstract

The expression of ABO antigens in human saliva is regulated by the *FUT2* gene, which encodes a secretor type α(1,2)fucosyltransferase. Secretors express ABO substrates in saliva and non-secretors do not. Secretor status is an object of concern, especially for susceptibility to various infectious diseases. A multitude of single nucleotide polymorphisms (SNPs) and copy number variations (CNVs) have been reported, and they show unique distributions among different populations. In this study, we selected 18 uncharacterized *FUT2* alleles listed in the Erythrogene database and obtained genomic DNA having these alleles. We experimentally confirmed the haplotypes, but 10 of 18 alleles disagreed with those in the database, which may be attributed to their low frequency. We then examined the activity of the encoded α(1,2)fucosyltransferase for 13 alleles by flow cytometry of H antigen expression. The impact of each nonsynonymous SNP on the enzyme was also estimated by software. We finally identified two non-secretor alleles (*se*^*610*^and *se*^*357,856,863*^) and one weak secretor allele (*se*^*262,357*^), while in silico analysis predicted that many alleles impair the function. The present results suggest that correct haplotyping and functional assays are desirable for analysis of the *FUT2* gene.

## Introduction

The human *FUT2* encodes the secretor type α(1,2)fucosyltransferase. This enzyme participates in synthesis of the H antigen, which is a precursor of A and B blood group antigens, on the surface of mucosa or in secretions. Secretor status is dominant over non-secretor status, and therefore the presence of at least one functional *FUT2* allele (*Se*) results in the secretor phenotype, while the non-secretors are homozygotes of the null alleles (*se*) and lack ABH antigens in saliva and other body fluids. Weak-secretors are homozygotes of the weak-secretor allele (*Se*^*w*^, see Table 1) or heterozygotes of *Se*^*w*^*/se* and express lower amounts of these antigens than secretors do^1,2^.

**Table 1:** Distribution of SNPs and alleles of *FUT2* in various populations.

Allele	SNPs	Amino acid changes	Populations
**Secretor alleles**			
*Se*		No change	Various populations
*Se*^*40*^	40A>G	I14V	Africans, Puerto Ricans
*Se*^*278,357*^	278C>T 357C>T	A93V	Iranians, Sri Lankans, Israelis
*Se*^*357*^	357C>T	No change	Various populations
*Se*^*357,480*^	357C>T 480C>T	No change	Africans, South and East Asians, Europeans
*Se*^*375*^	375A>G	No change	New Guineans, African, Samoans
*Se*^*400*^	400G>A	V134I	New Guineans, Samoans
*Se*^*481*^	481G>A	D161N	Africans
**Nonsecretor or weak-secretor alleles**			
*se*^*302*^	302C>T	I101P	South Asians, Peruvians
*se*^357,385^*	385A>T	I129F	East and Southeast Asians (50%)
*se*^*357,571*^	357C>T 571C>T	R191X	Samoans, Taiwanese
*se*^*357,480,778del*^	357C>T 480C>T 778delC	259fs275X	Africans
*se*^*428*^	428G>A plus 4	W143X	Europeans, Africans, West Asians (50%), South Asians, Latin Americans
*se*^*628*^	628C>T	R210X	Taiwanese
*se*^*841*^	841G>A	Q292R	Peruvians
*se*^*849*^	849G>A	W283X	Taiwanese

*Represents weak secretor allele (*Se*^*w*^).

The prevalence of non- or weak secretors is about 20–25% in most geographic regions, and statistical analysis of genetic variations of this locus suggest that the relative frequencies of secretors and non-secretors (or weak secretors) have been maintained for a long time by balancing selection, and that various pathogens may be considered an important selective force^3,4^.

The coding sequence of *FUT2* is not interrupted by an intron and locates solely in the second exon, which encodes a 343-amino acid protein. This makes it easy to determine the haplotypes of SNPs of the protein coding region^5,6^. Because we numbered the nucleotide positions of *FUT2* using a short coding region (999 bp: 332 amino acids) in previous studies^3,7^, we also used this to describe SNPs or alleles for easy comparison, while we used a long coding region (1032 bp: 343 amino acids) that has an additional 33 bp at the 5′-region for in silico analyses only to predict the impact of SNPs on the protein.

Because the blood group antigens on the surface of mucosa or in secretions can be used as a scaffold for infection by some kinds of pathogens, secretor status has been reported to be associated with susceptibility to viruses such as norovirus and rotavirus^8,9^. In addition, genome-wide association studies revealed its association with Crohn’s disease, celiac disease, and inflammatory bowel disease^10,11^.

Many single nucleotide polymorphisms (SNPs) and recombinant null alleles have been identified so far^4,12–16^. Representative SNPs and their alleles are shown in Table 1. Interestingly, some of them are distributed in a population-specific pattern. For association studies on secretor status, we need to know the genetic background of *FUT2* of the target populations unless we can determine the secretor status by phenotype. However, because amino acid substitutions do not always affect the activity of the encoded Se enzyme, predicting it is sometimes difficult^17–19^. To examine the impact of each of the SNPs on the enzyme activity, we can monitor the α(1,2)fucosyltransferase activity by transient expression in cultured cells, followed by measurement of the enzyme activity using ^14^C-labeled fucose or its acceptor or examination of the H antigen on the cell surface by flow cytometry^17,20,21^.

The 1000 Genome Project (https://www.internationalgenome.org/) is the largest public catalogue of human variation and genotype data^22^. Erythrogene v0.8 (27 Nov 2017) (http://www.erythrogene.com/) extracted the data of blood group alleles from 1000 Genomes and matched them against blood group reference lists^23^. For *FUT2*, 73 alleles are listed at present. To survey the uncharacterized nonfunctional alleles of *FUT2*, we selected 18 alleles listed in Erythrogene (80T>C; 357C>T; 385A>T, 357C>T; 403C>T; 950C>T,357C>T; 964A>G, 357C>T; 385A>T; 539G>A,357C>T; 610G>T, 40A>G; 357C>T; 616G>C , 357C>T; 715C>T, 357C>T; 856T>C; 863C>T, 370G>A, 58C>T; 357C>T, 357C>T; 566T>C, 418G>A; 863C>T, 357C>T; 980C>A, 357C>T; 385A>T; 980C>A, 357C>T; 544G>A; 771G>A, 205G>A; 357C>T, 357C>T; 542C>T, 262A>C; 357C>T) and obtained genomic DNA of 22 individuals who have these alleles with reference to Phase 3 of the 1000 Genomes Project (Table 2). In this study, we experimentally confirmed the nonsynonymous SNPs and their haplotypes (alleles). We also analyzed the encoded enzyme activities by transient expression assays. In addition, we predicted the effect of each nonsynonymous SNP on the protein by several in silico methods.

**Table 2:** DNA samples and relationship between *FUT2* alleles on database and experimentally determined.

Coriell No	Allele in database (Erythrogene)	Experimentally determined alleles		Attribution
		Target allele	Other allele	
HG00701*	80T>C; 357C>T; 385A>T	80T>C; 357C>T	*se*^*357,385*^	Southern Han Chinese
HG01121**	357C>T; 403C>T; 950C>T	357C>T; 950C>T	357C>T; 403C>T	Colombian in Medellin
HG01705	357C>T; 964A>G	357C>T; 964A>G	*Se*	Iberian populations in Spain
HG02130*	357C>T; 385A>T; 539G>A	357C>T; 539G>A	*se*^*357,385*^	Kinh in Ho Chi Minh City
HG02345*	357C>T; 610G>T	610G>T	*Se*^*357*^	Peruvian in Lima
HG03279*	40A>G; 357C>T; 616G>C	40A>G; 616G>C	*Se*^*357*^	Esan from Nigeria
HG03681	357C>T; 715C>T	357C>T; 715C>T	*se*^*del*^	Sri Lankan Tamil in the UK
HG03973	357C>T; 856T>C; 863C>T	357C>T; 856T>C; 863C>T	*se*^*428*^	Indian Telugu in the UK
HG04017***	370G>A	370G>A on *se*^*428*^	wild	Indian Telugu in the UK
NA11920	58C>T; 357C>T	58C>T; 357C>T	*Se*^*357*^	Utah residents CEPH with Northern and Western European ancestry
NA18626*	357C>T; 566T>C	566T>C	*Se*^*357*^	Han Chinese in Beijing
NA18908**	481G>A; 863C>T	481G>A	863C>T on *se*^*428*^	Yoruba in Ibadan
NA18951	357C>T; 980C>A	357C>T; 980C>A	*se*^*357,385*^	Japanese in Tokyo
NA18974				Japanese in Tokyo
NA19063				Japanese in Tokyo
NA19067*	357C>T; 385A>T; 980C>A	357C>T; 980C>A	*se*^*357,385*^	Japanese in Tokyo
NA19625***	357C>T; 544G>A; 771G>A	544G>A; 771G>A on *se*^*428*^	*Se*^*357*^	African ancestry in Southwest USA
NA20520	205G>A; 357C>T	205G>A; 357C>T	*se*^*428*^	Toscani in Italy
NA20531			*se*^*428*^	Toscani in Italy
NA20532			*Se*^*357*^	Toscani in Italy
NA20805	357C>T; 542C>T	357C>T; 542C>T	*se*^*428*^	Toscani in Italy
NA20891	262A>C; 357C>T	262A>C; 357C>T	*Se*^*357*^	Gujarati Indians in Houston

Note: Asterisks represents samples whose experimentally determined *FUT2* haplotype was different from that in the Erythrogene database.

*Functional analysis was performed.

**Functional analysis was not performed because it was already characterized.

***Functional analysis was not performed because target SNP was on *se*^*428*^.

## Results

### Sequence and haplotype determination of *FUT2*

To survey the nonfunctional alleles of *FUT2* in the database, we determined the DNA sequence of the total coding region of the *FUT2* gene of 22 individuals who were expected to have uncharacterized alleles according to Erythrogene^23^. We detected all of the registered SNPs without deficiency and excess in respective DNA samples in the database by direct Sanger sequencing of the *FUT2* coding region and encountered 17 uncharacterized nonsynonymous SNPs that have not been identified yet: 58C>T, 80T>C, 205G>A, 262A>C, 370G>A, 539G>A, 542C>T, 544G>A, 566T>C, 610G>T, 616G>C, 715C>T, 856T>C, 863C>T, 964A>G, and 980C>A. In addition to these, the alleles of 357C>T, 403C>T, and 950C>T were also investigated, although 403C>T and 950C>T themselves have been identified in Latin Americans (*Se*^*357,403*^) and a Mongolian (*se*^*357,950*^), respectively^17,19^. We then determined their haplotypes by subcloning them into plasmids. However, sequencing the clones revealed that the haplotypes of ten of 18 alleles were different from those registered in the database, even when the distance between SNPs was only 154 bp (positions 385 and 539 in HG02130, positions 216 (of *se*^*428*^) and 370 in HG04017, Table 2). Among the ten alleles, three uncharacterized nonsynonymous SNPs (370G>A, 544G>A, and 863C>T) were found on the nonfunctional allele *se*^*428*^, and 403C>T and 950C>T were found on another chromosome (Table 2). Accordingly, we performed functional analyses of the 13 alleles shown in Table 3. Haplotyping revealed that 863C>T was on two different alleles, 357C>T; 856T>C; 863C>T (HG03973 (Indian Telugu in the UK)) and 428G>A (plus 4 additional SNPs); 863C>T (NA18908 (Yoruba in Ibadan)) (Table 2).

**Table 3:** Evaluation of candidates for nonfunctional *FUT2* alleles by expression of cell surface H antigens or in silico analysis of the nonsynonymous SNP.

Allele	Expression level (± SD) n = 5	Luciferase activity ($\times$ 10^6^ RLU) (± SD) n = 5	Rs No	Amino acid change	MutationTester	MutationAssessor (score)	Polyphen2 ( score)	SIFT (score)	Allele
80T>C; 357C>T	25.79 (5.43)	2.33 (0.25)	rs199854717	M27T	P	N (-0.835)	B ( 0.000)	T (0.16)	*Se*^*80,357*^
357C>T; 964A>G	19.57 (2.34)	2.99 (0.19)	rs546134946	I322V	D	M (2.990)	P (0.961)	A (0.00)	*Se*^*357,964*^
357C>T; 539G>A	23.99 (3.69)	3.13 (0.11)	rs571045256	R180Q	P	N (0.620)	B ( 0.026)	T (0.29)	*Se*^*357,539*^
610G>T	0.63 (0.36)	4.45 (0.46)	rs373601796	G204W	D	M (3.005)	P (1.000)	A (0.00)	*se*^*610*^
40A>G; 616G>C	21.32 (2.75)	3.67 (0.39)	rs146094646	V206L	P	L (1.680)	B (0.172)	A (0.00)	*Se*^*40,616*^
357C>T; 715C>T	20.45 (7.38)	3.22 (0.39)	rs564523135	R239W	D	M (2.915)	P (1.000)	A (0.05)	*Se*^*357,715*^
357C>T; 856T>C; 863C>T	0.68 (0.27)	3.02 (0.50)							*se*^*357,856,863*^
**Alleles generated by mutagenesis**									
357C>T; 856T>C	0.63 (0.28)	1.53 (0.42)	rs537571802	Y286H	D	M (3.005)	P (1.000)	A (0.00)	
357C>T; 863C>T	7.98 (0.55)	2.31 (0.74)	rs146216418	T288M	P	L (1.445)	P (0.993)	A (0.00)	
58C>T; 357C>T	24.31 (3.82)	2.95 (0.24)	rs201010975	R20W	P	M (2.915)	P (1.000)	A (0.02)	*Se*^*58,357*^
566T>C	9.28 (0.99)	1.39 (0.35)	rs200698586	V189A	D	M (3.005)	P (1.000)	A (0.00)	*Se*^*566*^
357C>T; 980C>A	20.32 (2.06)	1.40 (0.27)	rs139058715	S327Y	P	M (1.995)	P (0.989)	A (0.00)	*Se*^*357,980*^
205G>A; 357C>T	19.27 (9.62)	2.19 (0.11)	rs143120145	A69T	D	M (3.005)	P (1.000)	A (0.00)	*Se*^*205,357*^
357C>T; 542C>T	21.69 (1.50)	1.75 (0.23)	rs138954645	P181L	D	M (2.810)	P (0.973)	A (0.00)	*Se*^*357,542*^
262A>C; 357C>T	2.03 (0.43)	2.58 (0.53)	rs532253708	M88L	P	M (3.005)	P (0.988)	A (0.00)	*se*^*262,357*^(*Se*^*w*^)

Luciferase activities are presented by Relative Light Unit (RLU). P and D represent polymorphism and disease-causing, respectively (MutationTester). N, M, and L represent neutral, medium and low, respectively (MutationAssessor). B and P represent benign and probably damaging, respectively (Polyphen2). T and A represent tolerated and affect protein function, respectively (SIFT).

### Functional analyses of candidates of non-secretor alleles

**Figure 1:**
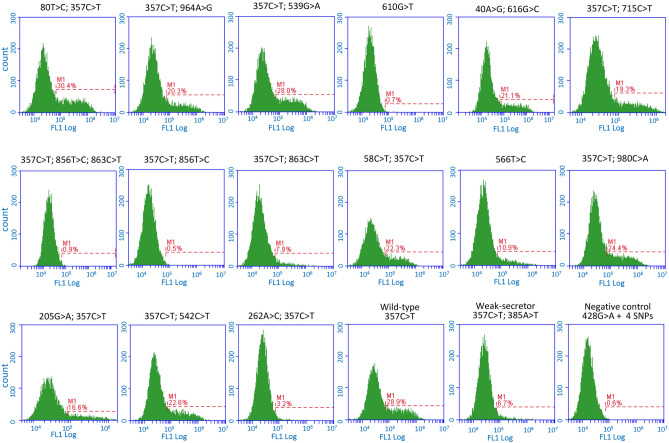
Expression of H antigens in the COS-7 cells transfected with various *FUT2* constructs. The *FUT2* allele containing uncharacterized SNP(s) of the *FUT2*, *Se*^*357*^ (wild-type allele), *se*^*357,385*^ (weak-secretor allele), or *se*^*428*^ (negative control) subcloned into pcDNA3.1(+) plasmids was transfected into COS-7 cells. After 2 days of culture, the cells were incubated with 1E3 antibody, followed by incubation with FITC-conjugated goat anti-mouse IgM secondary antibody, and expression of H antigen on the cell surface was monitored by flow cytometry.

To determine whether each uncharacterized *FUT2* allele encodes a functional Se enzyme or not, we ligated each allele into the mammalian expression vector pcDNA3.1, then transfected it into COS-7 cells. The cell surface H antigen was examined by flow cytometry using a monoclonal antibody to H type 1–4 (1E3)^24^. We performed five independent transient expression experiments, and the representative result for each allele was shown in Fig. 1. The expression levels of H antigens on cell surface are shown in Table 3. The percentage of H antigen–positive cells transfected with pcDNA3.1 ligated with the *FUT2* of 80T>C; 357C>T, 964A>G; 357C>T, 357C>T; 539G>A, 40A>G; 616G>C, 357C>T; 715C>T, 58C>T; 357C>T, 357C>T; 980C>A, 205G>A; 357C>T, and 357C>T; 542C>T were almost identical to those ligated with the positive control of *Se*^*357*^ (wild-type allele, 26.22 ± 5.06%). On the other hand, the percentage of pcDNA3.1 ligated with *FUT2* of 566T>C (9.28 ± 0.99%) was less than half of the positive control but higher than that of *se*^*357,385*^ (weak-secretor allele, 4.86 ± 0.76%), while that of 262A>C; 357C>T (2.03 ± 0.43%) was lower than that of *se*^*357,385*^. In the same experimental condition, expression of the H antigen on cells transfected with pcDNA3.1-*FUT2* of 610G>T or 357C>T; 856T>C; 863C>T was almost undetectable as was that of *se*^*428*^ (0.68 ± 0.19%, Table 3).

### Determination of causal SNP of FUT2-357C>T; 856T>C; 863C>T for inactivation of the encoded enzyme

As mentioned above, the expression study suggested that *FUT2* of 357C>T; 856T>C; 863C>T was a nonfunctional allele. However, this allele contained two nonsynonymous SNPs, 856T>C and 863C>T, and it was unclear which SNP was involved in inactivation of the Se enzyme. We generated *FUT2* of 357C>T; 856T>C and *FUT2* of 357C>T; 863C>T by in vitro mutagenesis and performed transient expression and flow cytometry. As shown in Table 3, 856T>C completely impaired and 863C>T somewhat impaired the activity.

### Estimation of significance of uncharacterized nonsynonymous SNPs

In addition to the transient expression study, we predicted the possible impacts of 14 amino acid substitutions on the functions of encoded proteins using four software programs.

The results of predictions were not always consistent with those of expression experiments (Table 3). When *FUT2* of 610G>T, of 357C>T; 856T>C; 863C>T, and of 262A>C; 357C>T are considered to be nonfunctional alleles and the others functional, the ratio of estimated mismatch was 42.9%, 64.3%, 57.1%, and 64.3% by MutationTaster, MutationAssessor, PolyPhen-2, and SIFT, respectively. Of 14 SNPs, the predicted effects were matched for all software and experiments for M38T, R191Q, G215W, and Y297H, while they were completely mismatched for I333V, R250W, V200A, A80T, and P192L (Table 3). The software generally tended to overestimate the impacts of the nonsynonymous SNPs we tested here.

## Discussion

Recently, public databases are available that provide genetic variation data, and websites dedicated to various purposes using these data are also accessible. The 1000 Genomes Project Phase 3 provides a list of variants and haplotypes of 2,504 individuals from 26 populations using a combination of low-coverage whole-genome sequencing, deep exome sequencing, and dense microarray genotyping^23^. One study reported that phasing and imputation for rare variants are unreliable, which likely reflects the limited sample size of the project data^25^. In this study, we observed discordance between the haplotypes on Erythrogene from the 1000 Genomes project and those experimentally phased haplotypes for ten out of 18 haplotypes. Actually, the *FUT2* alleles we investigated have frequencies of 0.02 to 0.06% in the studied 2504 samples of 26 populations on 1000 Genomes, that is, one to three of 5008 alleles. That may be the reason for the discordance, but it should not be ignored in association studies on secretor status when its prevalence is at a certain level in the target populations. Previously, we identified 400G>A (rs370886251) of a functional *FUT2* allele *Se*^*400*^ in a Samoan population with a frequency of 2.1% (one of 48 alleles)^26^. This allele was also observed in Indonesians at 1.2% (two of 166 alleles)^27^. In addition, we encountered it in six New Guinean populations with the relatively high prevalences of 10.6% (42 of 114 alleles) to 36.8% (7 of 66 alleles)^28^. These results suggest that 400G>A is distributed in Oceania and neighboring countries. However, 400G>A is not listed in 1000 Genomes and subsequently Erythrogene. These observations raise the possibility that some of the nonsynonymous SNPs we examined here may be present at unignorable frequencies in certain populations^25^.

The best way to evaluate the activity of the Se enzyme may be consideration in conjunction with phenotypic data using red blood cells or saliva as specimens. In the Lewis blood group, Le(a − b +) is identified as a secretor, Le(a + b −) as a non-secretor, and Le(a + b +) as a weak-secretor^29^. We can also measure the amount of ABO substances in saliva. However, we could not perform phenotyping and can only speculate on the secretor status based on the genetic information. In this study, we estimated the activity of the enzyme encoded by *FUT2* alleles of interest by transient expression and subsequent flow cytometry for detection of cell surface H antigens expressed. In addition, the impacts of the target missense SNPs on the Se enzyme are also deduced by four software programs because we experienced a discrepancy between the results obtained by in vitro and those by in silico analyses^17–19^. In the results, we also observed discrepancies for six to nine out of 14 SNPs, while all of the SNPs reside in the catalytic domain. Previously, we identified a *FUT2* allele with 357C>T; 685G>A in a Bangladeshi secretor individual with genotype of 357C>T; 685G>A/302C>T. The α(1,2)fucosyltransferase activity of the enzyme encoded by *FUT2* of 357C>T; 685G>A was 8% of that of the wild-type allele^26^*.* From this observation, we categorized *FUT2* of 566T>C as a secretor allele (*Se*^*566*^) and that of 262A>C; 357C>T as a weak secretor allele (*se*^*262,357*^*, Se*^*w*^), while the secretor status phenotypes were unknown and the 566T>C substitution seemed to partially impair enzyme activity (Table 3).

In this study, we used an academically isolated monoclonal antibody, but the H antigens could be evaluated by the commercially available UEA-1 lectin (*Ulex europaeus* agglutinin I)^19,21,24^. In addition, as mentioned earlier, the sequence of the *FUT2* is coded solely in exon 2. This makes it easy to clone the coding sequence or determine the haplotype of the coding polymorphisms.

Considering all the factors mentioned above, experimental procedures such as the haplotyping by cloning and evaluation of activity of the Se enzyme are desirable for *FUT2*. However, the 1000 Genome Project provides a valuable catalogue of human genetic variation, and the Erythrogene focus on blood group systems also enables easy reference to the *FUT2* alleles from the 1000 Genome Project. Actually, the existences of listed SNPs themselves were completely confirmed in the indicated samples. In addition, the software programs for prediction of the functional impacts of amino acid substitutions are valuable tools, especially for the proteins whose functions are hard to estimate experimentally. Accordingly, there is no doubting the helpfulness of these databases or software programs.

## Conclusion

We identified two non-secretor alleles (*se*^*610*^and *se*^*357,856,863*^) and one weak secretor allele (*se*^*262,357*^) in samples in the 1000 Genome Project. Experimental phasing and expression studies are desirable for analysis of *FUT2*.

## Materials and methods

The study protocol was approved by the ethical committees of Kurume University School of Medicine (No. 342).

### DNA samples

Twenty-two genomic DNAs (HG00701, HG01121, HG01705, HG02130, HG02345, HG03279, HG03681, HG03973, HG04017 of the 1000 Genomes project, NA11920 of CEPH/Utah Pedigree 1423, NA18626, NA18908, NA18951, NA18974, NA19063, NA19067, NA19625, NA20520, NA20531, NA20532, NA20805, NA20891of International HapMap Project) were purchased from the Coriell Institute for Medical Research (Camden, NJ, USA) (Table 2).

### PCR amplification of coding region of *FUT2* and sequence analysis

The coding regions of *FUT2* were amplified and directly sequenced as described previously^17^.

### Haplotype determination of *FUT2*

To determine the haplotypes of individuals who were heterozygous at two or more positions, we cloned PCR products into a plasmid and sequenced the clones as described previously^17^.

### Transient expression study and evaluation of impact of nonsynonymous SNPs of *FUT2*

To evaluate the significance of each of uncharacterized allele, a transient expression experiment was performed as described previously^17^. The *FUT2* alleles containing each SNP concerned, 357C>T (*Se*^*357*^: wild-type allele), 171A>G and 216C>T and 428G>A and 739G>A and 960A>G (*se*^*428*^: nonsecretor allele) or 357C>T and 385A>T (*se*^*357,385*^: weak-secretor allele) were inserted into pcDNA3.1(+) vectors. Three μg of each construct together with 100 ng of the pGL3 Promoter was transfected into 5 × 10^5^ COS-7 cells (African green monkey kidney fibroblast-like cell) by means of TransIT-X2 (Mirus Bio LLC, Madison, WI).After 2 days of culture, the cells were incubated with 1E3 antibody for H type 1–4, followed by incubation with FITC-conjugated goat anti-mouse IgM (Bethyl Laboratories, Montgomery, TX) secondary antibody, and expression of H antigen on the cell surface was monitored by flow cytometry (BD Accuri C6, Becton Dickinson, Franklin Lakes, NJ) as described previously^18,24^. The experiments were performed five times. About 1 × 10^5^ cells were lysed and the firefly luciferase activity was assayed using ONE-Glo Luciferase Assay System (Promega, Madison, WI) and the similar transfection efficiency in each experiment was confirmed by the intensity of luciferase light (Table 3).

### Prediction of impacts of nonsynonymous SNPs on Se enzyme

The effect of each nonsynonymous SNP of *FUT2* on the function of the enzyme were predicted using four free software programs, MutationTaster (http://www.mutationtaster.org/)^30^, MutationAssessor (http://mutationassessor.org/r3/)^31^, PolyPhen-2 (http://genetics.bwh.harvard.edu/pph2/)^32^, and Sorting Intolerant From Tolerant (SIFT) (http://sift.jcvi.org/)^33^.

### Introduction of reverse substitution in allele containing 357C>T; 856T>C; 863C>T

Because functional analysis showed one allele containing 357C>T; 856T>C; 863C>T to be a nonfunctional allele, site-specific mutagenesis of the plasmid DNA was performed using a Q5 Site-Directed Mutagenesis Kit (New England Biolabs Japan Inc., Tokyo, Japan) to determine which substitution is responsible for inactivation of the enzyme. The primer set used for reverse substitution at 856 (C to T) was 5′-CTG GGC CGC ATA CCT CAT GGG-3′ (forward primer, 846–866 bp of *FUT2*, position 856 and 863 are underlined) and 5′-ATC CCG AAC GTC CCA ATG GTC ATG-3′ (reverse primer, 822–845 bp of *FUT2*). That for 863 (T to C) was 5′-GCA CAC CTC ACG GGC GGA GAC-3′ (forward primer, 853–873 bp, position 856 and 863 are underlined) and 5′-GGC CCA GAT CCC GAA CGT-3′ (reverse primer, 835–852 bp of *FUT2*). The plasmid pcDNA3.1(+) containing *FUT2* of 357C>T; 856T>C; 863C>T was used as a template. All procedures were performed according to the manufacturer’s protocol. The cycling conditions were an initial denaturation at 98 °C for 30 s, followed by 25 cycles of denaturation at 98 °C for 10 s, annealing at 70 °C (for 856 reverse substitution) or 72 °C (for 863 reverse substitution) for 20 s, and extension at 72 °C for 3 min, followed by final extension at 72 °C for 2 min. The sequences of the isolated clones were confirmed. Finally, we obtained two constructs, pcDNA3.1(+) containing *FUT2* of 357C>T; 856T>C and 357C>T; 863C>T.
